# Mapping of three novel linear B-cell epitopes on the VP7 protein of epizootic hemorrhagic disease virus with monoclonal antibodies

**DOI:** 10.1186/s13567-026-01842-7

**Published:** 2026-09-19

**Authors:** Xinbing Hu, Yunru Zhong, Yingjuan He, Mingxin Zhang, Fanhua Meng, Yu Zhang, Zhancheng Tian, Guiquan Guan, Jijun He, Youjun Shang, Jun Ai, Junzheng Du

**Affiliations:** 1 State Key Laboratory of Animal Disease Control and Prevention, Lanzhou Veterinary Research Institute, College of Veterinary Medicine, Chinese Academy of Agricultural Sciences, Lanzhou University, Lanzhou, 730000 China; 2 Gansu Province Research Center for Basic Disciplines of Pathogen Biology, Lanzhou, 730046 China; 3 Animal Quarantine Laboratory, Technology Center of Kunming Customs, Kunming, 650200 China

**Keywords:** Epizootic hemorrhagic disease virus, VP7 protein, monoclonal antibodies, B-cell epitope mapping

## Abstract

Epizootic hemorrhagic disease virus (EHDV) is an important *Orbivirus* transmitted by *culicoides* midges. EHDV poses a significant threat to ruminant production worldwide. The VP7 protein is a highly conserved, group-specific antigen of EHDV, which serves as a key target for serological diagnosis. In this study, the recombinant VP7 (r-VP7) protein of EHDV-1 was expressed in an *Escherichia coli* expression system and used to immunize BALB/c mice. Four hybridoma cell lines secreting monoclonal antibodies (mAbs) against VP7 were successfully generated by the hybridoma technique, and named 5D1, 6A7, 7B11, and 7C4. Indirect ELISA, western blot analysis, and immunofluorescence assays demonstrated that all four mAbs specifically recognized both the r-VP7 protein and the native VP7 protein in EHDV-1-infected BHK-21 cells, with favorable reactivity and specificity. The VP7 protein was progressively truncated and expressed as a series of GST fusion proteins, and the linear B-cell epitopes recognized by these mAbs were precisely identified by western blotting. The results showed that 5D1 and 7B11 recognized the epitope ^83^DYIQNLATIGVLATPEI^99^, 7C4 recognized ^121^PDRQPFGYFL^130^, and 6A7 recognized ^229^APVNVNNPGQ^238^. Sequence alignment and cross-reactivity assays revealed that the three epitopes were highly conserved among EHDV serotypes and showed no cross-reactivity with the VP7 proteins of African horse sickness virus (AHSV) or bluetongue virus (BTV). Three-dimensional structural analysis indicated that all three epitopes were exposed on the surface of the VP7 trimer and were located in distinct structural domains. In summary, this study successfully generated four specific mAbs against the EHDV VP7 protein and identified three novel linear B-cell epitopes, providing a foundation for the development of specific EHDV serological diagnostic methods and epitope-based vaccines.

## Introduction

Epizootic hemorrhagic disease (EHD) is a vector-borne, infectious, non-contagious viral disease. It mainly affects wild and domestic ruminants, such as white-tailed deer, sheep, and cattle [1]. The virus is transmitted via bites of *Culicoides* midges [2]. White-tailed deer, which develop endothelial swelling, hemorrhages, and necrosis after infection, are the most affected species and commonly experience high mortality rates. The symptoms in cattle infected with Epizootic hemorrhagic disease virus (EHDV) are generally less severe than those in white-tailed deer; however, outbreaks among cattle have shown a trend toward increased virulence in recent years. In particular, infections caused by EHDV-6 and EHDV-7 have shifted from subclinical to clinical symptoms [3, 4]. Infected cattle experience fever, anorexia, dysphagia, swollen eyelids, dyspnea, conjunctival hyperemia and edema, congestion, and hemorrhage of the teats and udder, as well as lameness [4, 5]. To date, seven serotypes of EHDV have been officially recognized, and several additional putative serotypes have been reported, including isolates from China, Japan, and South Africa [5–9].

EHDV was first identified in white-tailed deer in New Jersey, USA, in 1995 and can cause high mortality in that species [1]. In 1959, an outbreak of Ibaraki disease in Japan was caused by the EHDV-2 serotype, resulting in severe clinical symptoms in infected cattle [10, 11]. During 1967−1970, EHDV was isolated from *Culicoides* in Nigeria [12]. Between 1982 and 2003, EHD outbreaks were reported successively in multiple regions worldwide, including the United States, Sudan, Canada, Indonesia, Australia, Mexico, South Africa, Japan, and Réunion Island [5]. Over the past 20 years, neighboring European countries such as Turkey, Israel, and Tunisia have successively reported outbreaks of EHDV serotypes 1, 6, and 7, whereas those regions were previously considered disease-free [4, 13, 14]. In 2022, EHDV was first detected in Europe, with outbreaks affecting cattle, deer, and sheep reported in Italy and southern Spain. The virus was identified as serotype 8, showing over 99% genetic similarity to the EHDV-8 strain that had been circulating in Tunisia since 2021 [15, 16]. By the summer of 2023, it had spread throughout Spain, reached Portugal, and expanded to France in September 2023. In the summer of 2024, new outbreaks were reported again in Spain, Portugal, and France [17].

Between 2014 and 2019, a serological survey of 116 regions across 15 provinces in China, involving 18 122 animal serum samples, detected EHDV antibodies in all provinces except Tibet. Significantly higher seropositivity rates were observed in southern provinces compared to Northern provinces [18]. A separate survey conducted in Guangdong province from 2013 to 2017, included 2886 bovine serum samples and reported an overall seropositivity rate of 57.87% [19]. In 2018, researchers isolated a novel serotype strain (YNDH/V079/2018) from cattle in Yunnan province, and between 2023 and 2024, four additional serotypes (EHDV-1, −5, −6, and −7) were isolated from Culicoides midges in the same province [8, 20]. To date, the EHDV serotypes reported in China include EHDV-1, −2, −5, −6, −7, −8, and −10, and all of which except EHDV-2 have been successfully isolated as viral strains [6].

EHDV belongs to the family *Sedoreoviridae*, genus *Orbivirus,* and shares many morphological and structural characteristics with the other members of the genus, in particular, bluetongue virus (BTV) [21, 22]. The virus is non-enveloped, with complete viral particles measuring approximately 80 nm, exhibiting icosahedral symmetry and a typical double-layered capsid structure [23]. The genome consists of ten linear double-strand RNA segments, encoding seven structural proteins (VP1-VP7) and five non-structural proteins (NS1, NS2, NS3/NS3a, NS4, NS5) [24–26]. The virion is composed of two layers of capsid. The outer capsid is made of VP2 and VP5 proteins, and the inner capsid is made of VP7 and VP3 proteins. The VP3 protein encases the viral transcriptase complex (VP1: RNA-dependent RNA polymerase, VP4: capping enzyme, VP6: helicase) and genomic RNA [27–29]. VP2 is a cellular receptor-binding protein that can induce the production of virus neutralizing antibodies. VP5, together with VP2, play key roles during the early stages of infection, being involved in virus attachment and entry into host cells [30, 31]. VP3 is responsible for the fundamental structure of the virion, and determines the proper assembly of the capsid [32]. VP7 is the immunodominant serogroup specific protein and its amino acid sequence is highly conserved among EHDV serotypes [33]. Non-structural proteins NS1, NS2, and NS3 are primarily involved in genome packaging, intracellular transport, capsid assembly, and virus release, whereas NS4 and NS5, primarily regulate host immune responses and maintain an intracellular environment conducive to virus replication [26, 34–39].

Since the VP7 protein is highly conserved across different serotypes; detection methods based on this protein can be used for the diagnosis of EHDV. ELISA, owing to its advantages of rapidity and high throughput, is recommended by the World Organization for Animal Health (WOAH) for the detection of EHDV antibodies in ruminant serum [40, 41]. In this study, the VP7 protein was expressed and purified using an *Escherichia coli* expression system, and then used to immunize BALB/c mice to generate mAbs against the EHDV VP7 protein. Then, the overlapping peptide method was used to map the B cell epitopes recognized by the monoclonal antibody, and the conservation characteristics of the epitopes among different EHDV serotypes and related orbiviruses was analyzed. The identified epitopes can serve as potential targets for epitope-based detection methods in serological diagnosis; their conservation across different serotypes offers insight into the structure and function of the VP7 protein. Furthermore, as a serogroup-specific antigen of EHDV, VP7 is highly conserved across serotypes and has been recognized as a promising target for vaccine design [42]. In conclusion, the VP7 B-cell epitopes identified in this study provide a basis for optimizing EHDV serological diagnostic methods, studying the function of the VP7 protein, and developing epitope vaccines.

## Materials and methods

### Cell, animal, virus, serum, proteins

SP2/0 mouse myeloma cells were cultured in RPMI 1640 (Gibco, Waltham, USA) with 20% fetal bovine serum (FBS; Gibco, Waltham, USA). BHK-21 cells were cultured in Dulbecco’s Modified Eagle’s Medium (DMEM; Gibco, Waltham, USA) with 5% FBS. Both cell lines were cultured at 37 °C in a humidified 5% CO₂ atmosphere. The Laboratory Animal Center of Lanzhou Veterinary Research Institute, CAAS, provided BALB/c mice. The EHDV-1 strain, EHDV-positive serum, recombinant VP7 of African horse sickness virus serotype 1 (AHSV-1), and recombinant VP7 of Bluetongue virus serotype 1 (BTV-1) expressed in *E. coli* were stored in our laboratory.

### Expression and identification of recombinant VP7 protein

The full-length gene sequence of EHDV-1 VP7 (GenBank accession number: OM953797.1) was codon-optimized for *E. coli*, then synthesized and cloned into the pET28a expression vector by GeneCreate Biological Engineering Co., Ltd (Wuhan, China). The recombinant plasmid was named pET28a-EHDVVP7 and transformed into BL21(DE3) competent cells (TransGen Biotech, Beijing, China). Isopropyl β-D-1-thiogalactopyranoside (IPTG, 1 mM Solarbio, Beijing, China) was added to induce expression at 37 °C for 6 h, during which samples were taken every hour for sodium dodecyl sulfate–polyacrylamide gel electrophoresis (SDS-PAGE) analysis. The induced samples were purified by Ni–NTA (GenScript, Nanjing, China) affinity chromatography and analyzed by SDS-PAGE. The purified VP7 protein was identified by western blotting using an anti-6 × His tag monoclonal antibody (Proteintech, Wuhan, China) and EHDV-positive serum as the primary antibodies, respectively.

### Preparation of monoclonal antibodies against VP7

Six-week-old BALB/c mice were immunized with recombinant VP7 (r-VP7) protein. For the first immunization, the r-VP7 protein was mixed well with an equal volume of complete Freund’s adjuvant (Sigma-Aldrich, St. Louis, MO, USA) and injected under the skin at several points on the back at a dose of 50 μg per mouse. Subsequently, at two-week intervals, the mice were boosted with a mixture of an equal volume of Freund's incomplete adjuvant (Sigma-Aldrich, St. Louis, MO, USA) and r-VP7. After the third immunization in mice, serum antibody titers were determined by indirect ELISA. The mouse with the highest antibody titer was selected and given an intraperitoneal booster injection of 50 μg of r-VP7. Three days later, mouse splenocytes were harvested and fused with SP2/0 myeloma cells using polyethylene glycol (Sigma-Aldrich, St. Louis, MO, USA). The fused cells were cultured in 96-well plates with HAT (Sigma-Aldrich, St. Louis, MO, USA) medium for 7 days, and then cultured in HT (Sigma-Aldrich, St. Louis, MO, USA) medium. When hybridoma cell clones grew to cover approximately one-third of the well bottom, the supernatants were collected and positive hybridoma cells were screened by indirect ELISA. Wells showing OD450 values at least 2.1-fold higher than the negative control were considered positive. The positive hybridoma cells were subsequently subcloned by limiting dilution, and the process was repeated three times to obtain stable monoclonal cell lines secreting specific antibodies.

### Indirect ELISA

Purified r-VP7 was diluted in carbonate-bicarbonate buffer (0.05 M, pH 9.6, containing sodium carbonate and sodium bicarbonate, Sigma-Aldrich, St. Louis, MO, USA) to 0.5 μg/mL, coated onto 96-well plates (Corning, NY, USA) at 100 μL per well, and incubated overnight at 4 ℃. Following three washes with PBST (phosphate-buffered saline containing 0.05% (V/V) Tween-20), the plates were blocked with 5% (W/V) skimmed milk in PBST at 200 μL per well and incubated at 37 °C for 1 h. After washing three times with PBST, hybridoma cell supernatants were added to the 96-well plates and incubated at 37 °C for 1 h. The plate was then washed again, and 100 μL of HRP-conjugated goat anti-mouse IgG (Abcam, Cambridge, UK) diluted at 1:100 000 in PBST was added to each well, followed by incubation at 37℃ for 1 h. After washing three times with PBST, 100 μL of TMB chromogenic solution (Surmodics, Eden Prairie, MN, USA) was added to each well, followed by incubation at 37 °C in the dark for 10 min. Then, 100 μL of stop solution (2 M H_2_SO_4_) was added to each well, and the absorbance at 450 nm was measured using a Multiskan SkyHigh microplate reader.

### Western blotting

To verify the reactivity of the mAbs with the VP7 protein, r-VP7 protein and EHDV-1-infected BHK-21 cells were separately mixed with loading buffer, separated by 12% SDS-PAGE, and then transferred onto a PVDF membrane (Merck, Darmstadt, Germany) using transfer buffer (25 mM Tris, 192 mM glycine, 20% methanol). The membrane was blocked with 5% skimmed milk at room temperature for 1 h. The hybridoma cell supernatant was then added and incubated at room temperature for 1 h. Subsequently, the membrane was washed three times with PBST for 10 min each, followed by the addition of HRP-conjugated goat anti-mouse IgG (diluted at 1:10 000 in PBST with 5% skimmed milk) and incubated at room temperature for 1 h. The membrane was washed three times with PBST, and the protein bands were visualized using enhanced chemiluminescence (ECL) reagents (ThermoFisher, Waltham, MA, USA). Images were captured with a ChemiDoc XRS+ Imaging System.

### Immunofluorescence assay

BHK-21 cells were pre-seeded in cell culture plates. When the cells reached 70%–90% confluence they were infected with EHDV-1 at an MOI of 0.1. After incubation at room temperature for 1 h, the inoculum was discarded, and 500 μL of DMEM supplemented with 2% FBS was added for further culture. At 24 h post-infection, the culture supernatant was removed, and the cells were fixed with 4% paraformaldehyde (Servicebio, Wuhan, China) at room temperature for 1 h. After three washes with PBS, the cells were permeabilized with 0.5% Triton X-100 (diluted in PBS, Solarbio, Beijing, China) at room temperature for 10 min. Following three washes with PBS, the cells were blocked with 3% BSA (diluted in PBS, Solarbio, Beijing, China) at room temperature for 1 h. Then, hybridoma supernatant against the EHDV VP7 protein was added and incubated at room temperature for 1 h. After three washes with PBS, Alexa Fluor 488-conjugated goat anti-mouse IgG (diluted 1:1000, Abcam, Cambridge, UK) was added and incubated at room temperature for 1 h. The cells were washed three times with PBS, and the nuclei were stained with Hoechst 33342 (diluted to 1 μg/mL in PBS, ThermoFisher, Waltham, MA, USA) for 10 min. After three washes with PBS, the cells were observed and imaged using an inverted fluorescence microscope.

### Mapping of the linear B cell epitopes of VP7 protein

**Figure 1:**
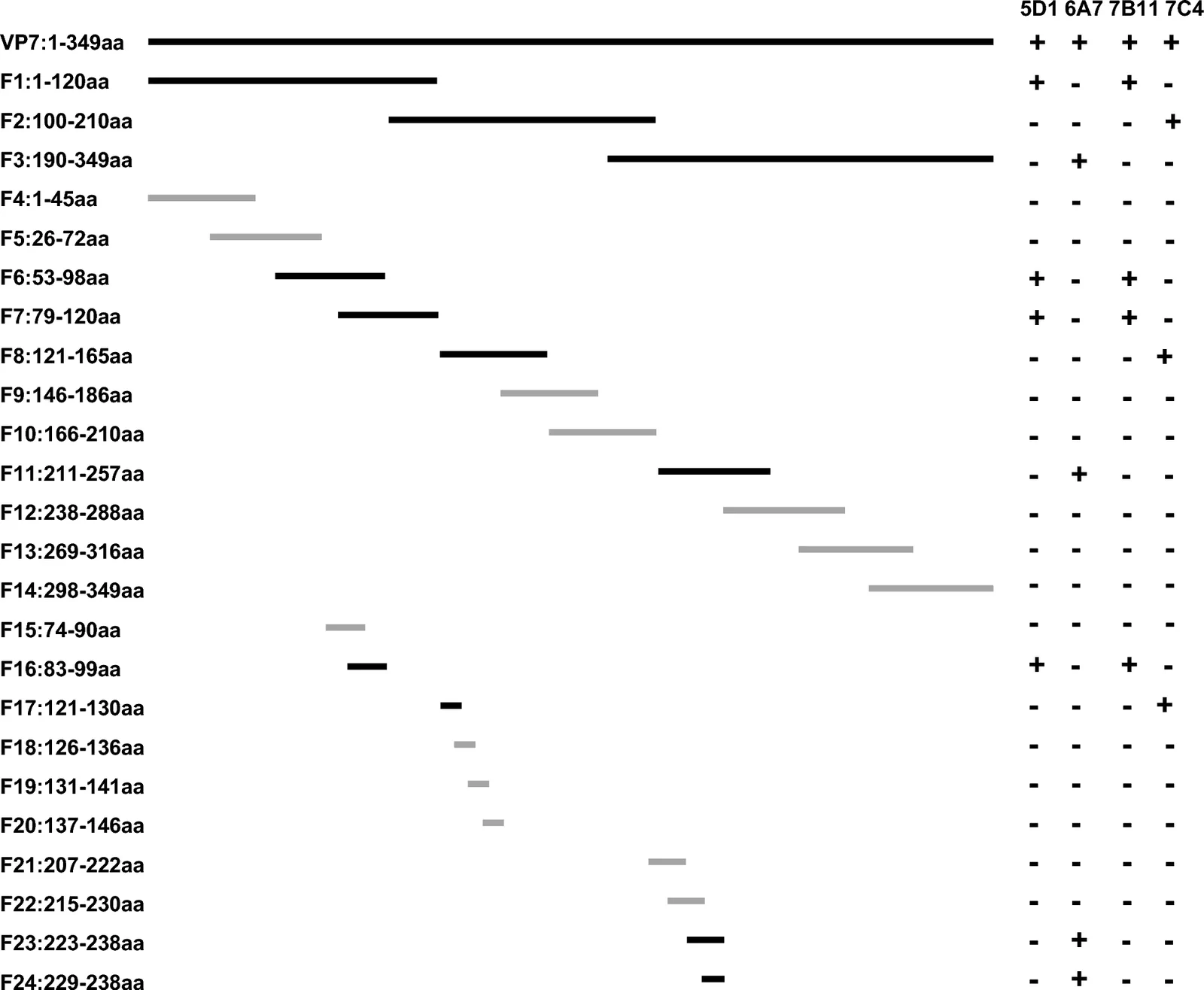
**Schematic diagram of truncated VP7 fragments for B-cell epitope mapping.** The segments that could be recognized by mAbs 5D1, 6A7, 7B11, and 7C4 are shown in black. The gray segments are those that none of the mAbs could identify.

To identify the epitope recognized by the mAbs, the EHDV VP7 gene was progressively truncated into a series of fragments, which were then cloned into the pGEX-6p-1 expression vector via *Eco*R I and *Xho* I restriction sites by Tsingke Biotechnology Co., Ltd. (Beijing, China) (Figure 1). The recombinant plasmids were transformed into BL21(DE3) competent cells for protein expression. The fusion proteins were first verified using a GST antibody (Proteintech, Wuhan, China), and the reactivity of the mAb with each truncated protein was detected by western blotting.

### Cross-reactivity between mAbs and *Orbivirus* VP7 protein

To evaluate the conservation of the identified epitopes, the amino acid sequences of VP7 proteins from all serotypes of EHDV, as well as from AHSV-1 and BTV-1, were downloaded from GenBank. Multiple sequence alignment of the sequences was performed using BioEdit software to analyze the conservation of the epitope regions among different EHDV serotypes and orbiviruses. Using PDB ID 1BVP as a template, the three-dimensional structure of EHDV VP7 protein was modeled using SWISS-MODEL. The model was visualized using PyMOL software to map the spatial distribution of the identified epitopes. In addition, western blotting was employed to detect the cross-reactivity of the mAbs with VP7 proteins of AHSV and BTV, further verifying the conservation of the epitope among different orbiviruses.

## Results

### Expression and identification of VP7 protein

**Figure 2:**
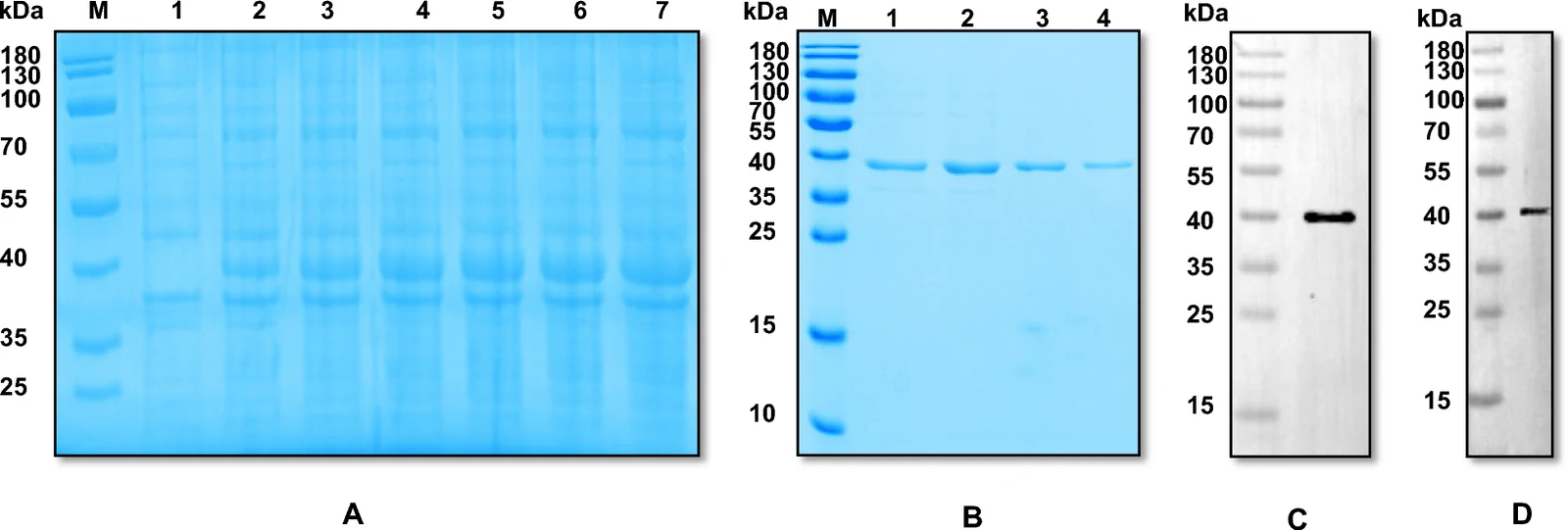
**Expression, purification, and identification of recombinant EHDV VP7 protein.** **A**: SDS-PAGE analysis of induced expression of recombinant VP7 protein. Lane 1: Uninduced bacteria; lane 2–7: Bacteria induced for 1–6 h. **B**: SDS-PAGE analysis of purified recombinant VP7 protein. **C**: Western blot detection of recombinant VP7 protein with anti-6×His mAb. **D**: Identification of recombinant VP7 protein by EHDV-positive serum.

The recombinant plasmid, pET28a-EHDV-VP7, was transformed into BL21(DE3) competent cells, and the expression of r-VP7 protein was induced at 37 ℃. Samples were collected at 1-h intervals for SDS-PAGE analysis. The results showed that the r-VP7 protein was successfully expressed in *E. coli*, with a molecular weight of approximately 40 kDa, consistent with the expected size (Figure 2A). Subsequently, the r-VP7 protein was purified by Ni–NTA affinity chromatography, and SDS-PAGE revealed a single band for the purified protein (Figure 2B). Western blot analysis demonstrated that the purified VP7 protein could be specifically recognized by the anti-His tag mAb (Figure 2C) and EHDV-positive serum (Figure 2D).

### Preparation and characterization of mAbs

**Figure 3:**
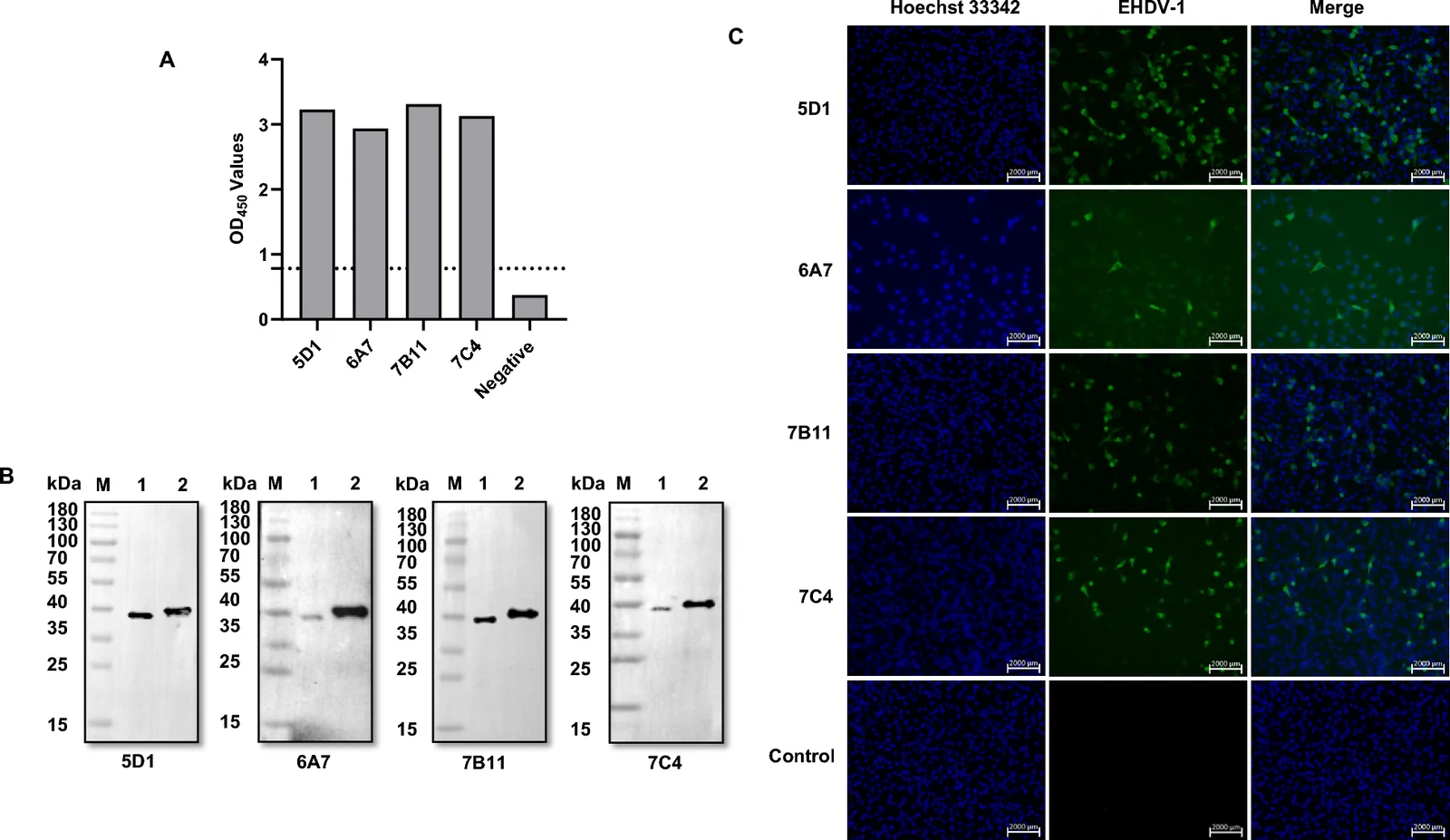
**Characterization of mAbs 5D1, 6A7, 7B11, and 7C4 against VP7.** **A** Indirect ELISA analysis of mAbs reactivity. **B** Western blot analysis of the mAbs (lane M: protein molecular weight markers; lane 1: EHDV-1-infected BHK-21 cells; lane 2: r-VP7 protein). HRP-conjugated goat anti-mouse IgG was used as the secondary antibody. **C** IFA analysis of the mAbs. BHK-21 cells infected with EHDV-1 were subjected to IFA at 24 h post-infection. Alexa Fluor488-conjugated goat anti-mouse IgG was used as a secondary antibody.

Hybridoma cell supernatants were screened by indirect ELISA, and four hybridoma cell lines stably secreting mAbs against EHDV VP7 protein were obtained, named 5D1, 6A7, 7B11 and 7C4 (Figure 3A). Western blot analysis showed that the four mAbs reacted specifically with r-VP7 protein, and also recognized native VP7 protein in EHDV-1-infected BHK-21 cells (Figure 3B). Notably, the r-VP7 protein migrated slower than the native VP7 protein, which was due to the presence of His tags and extra vector‑derived sequences carried by the recombinant protein. Immunofluorescence assays revealed that the four mAbs bound to EHDV-1-infected BHK-21 cells, with distinct green fluorescence observed, whereas no fluorescence signal was observed in the negative control (Figure 3C). Those results indicated that the four mAbs specifically recognized the EHDV VP7 protein with good reactivity and specificity.

### Epitope mapping of VP7 protein

**Figure 4:**
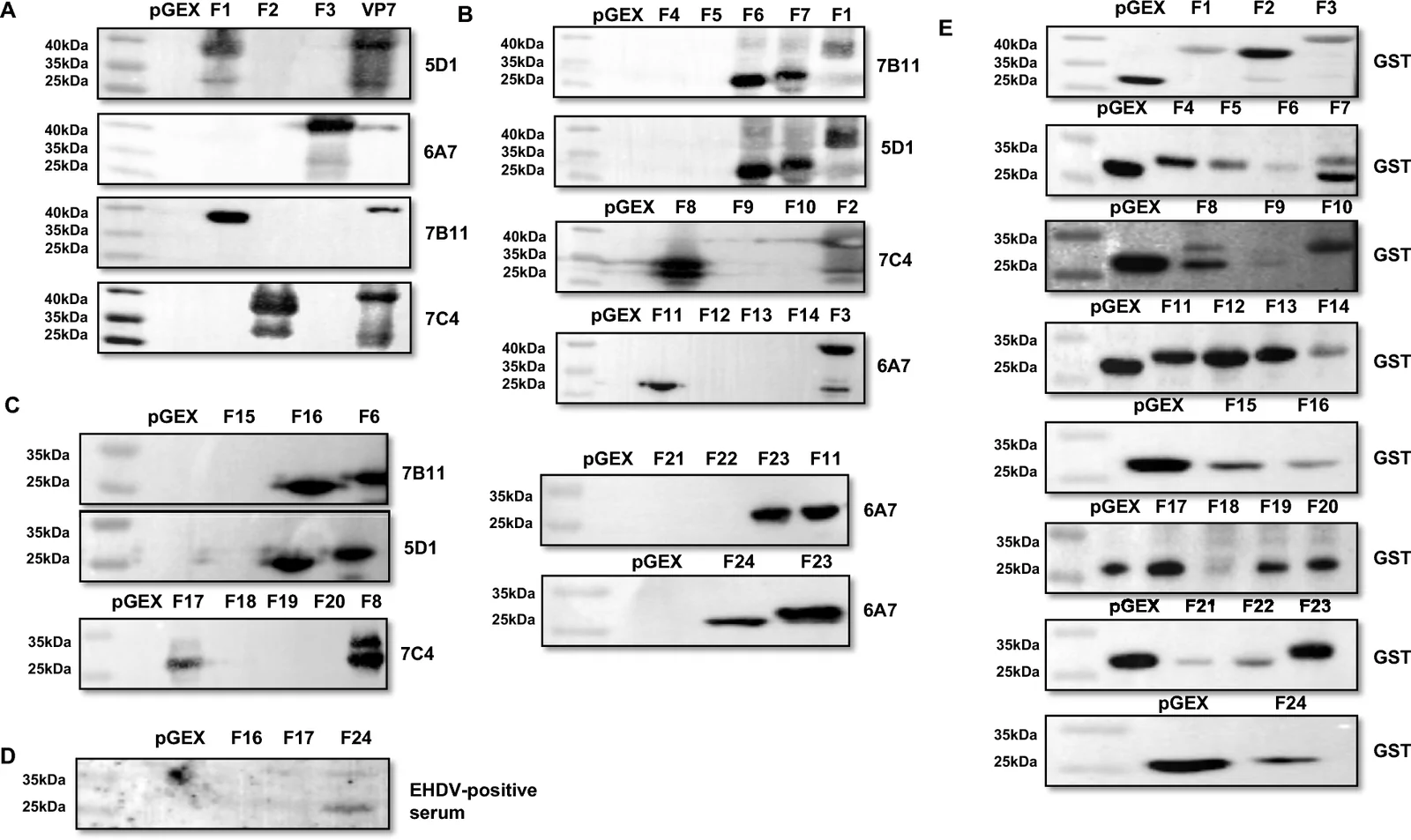
**Mapping of linear B-cell epitopes on EHDV VP7 recognized by mAbs and positive serum.** **A**: Reactivity of mAbs with the primary truncated VP7 fragments. Western blot analysis revealed that mAbs 5D1 and 7B11 recognized the F1 fragment, 7C4 recognized the F2 fragment, and 6A7 recognized the F3 fragment. **B**, **C**: Fine mapping of epitopes. The fragments F1, F2, and F3 were further truncated, expressed, and analyzed by western blotting. The results revealed that mAbs 5D1 and 7B11 recognized the epitope ^83^DYIQNLATIGVLATPEI^99^, mAb 7C4 recognized the epitope ^121^PDRQPFGYFL^130^, and mAb 6A7 recognized the epitope ^229^APVNVNNPGQ^238^. **D**: Reactivity of EHDV-positive serum with VP7 truncated fragments. Western blot analysis showed that only fragment F24 was specifically recognized. **E**: Confirmation of GST-fused VP7 truncated protein expression. pGEX, pGEX-6p-1 empty vector.

To precisely identify the antigenic epitopes recognized by the mAbs, the full-length EHDV VP7 protein was first truncated into three overlapping fragments, named F1 (1–120 aa), F2 (100–210 aa), and F3 (190–349 aa), respectively. Western blot analysis revealed that mAbs 5D1 and 7B11 specifically recognized the F1 fragment, 7C4 reacted with the F2 fragment, and 6A7 bound to the F3 fragment (Figure 4A). To further map the antigenic epitopes recognized by the four mAbs, a series of shorter truncated fragment were designed and constructed based on the F1, F2, and F3 fragments. Their reactivities were verified by western blotting. Through this stepwise truncation strategy, it was determined that mAbs 5D1 and 7B11 recognized fragment F16 (83−99 aa), mAb 7C4 recognized fragment F17 (121−130 aa), and mAb 6A7 recognized fragment F24 (229−238 aa) (Figure 4B, C). Subsequently, the identified epitopes were analyzed using EHDV-positive serum, and the results revealed that fragment F24 could be recognized by EHDV-positive serum (Figure 4D). Before epitope mapping, expression of the GST-fused VP7 truncated proteins was confirmed by western blot analysis using an anti-GST antibody (Figure 4E).

### Homology and cross-reactivity analysis

**Figure 5:**
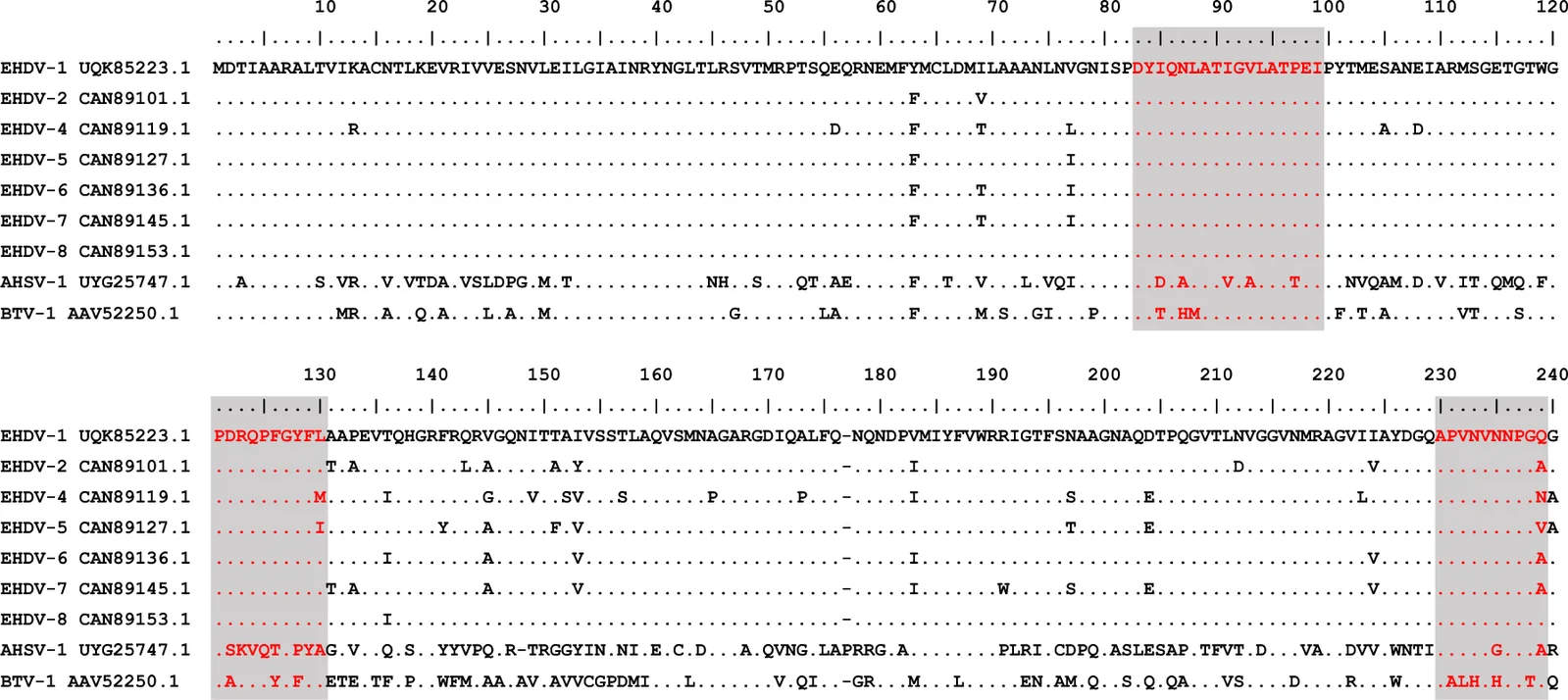
**Sequence alignment of the three epitope regions.** Amino acid sequences of VP7 proteins from EHDV serotypes, AHSV-1, and BTV-1 were aligned. These epitopes were highly conserved among EHDV serotypes, but were not conserved in AHSV or BTV VP7 proteins. The three identified epitopes are shown in red on the light gray background.

**Figure 6:**
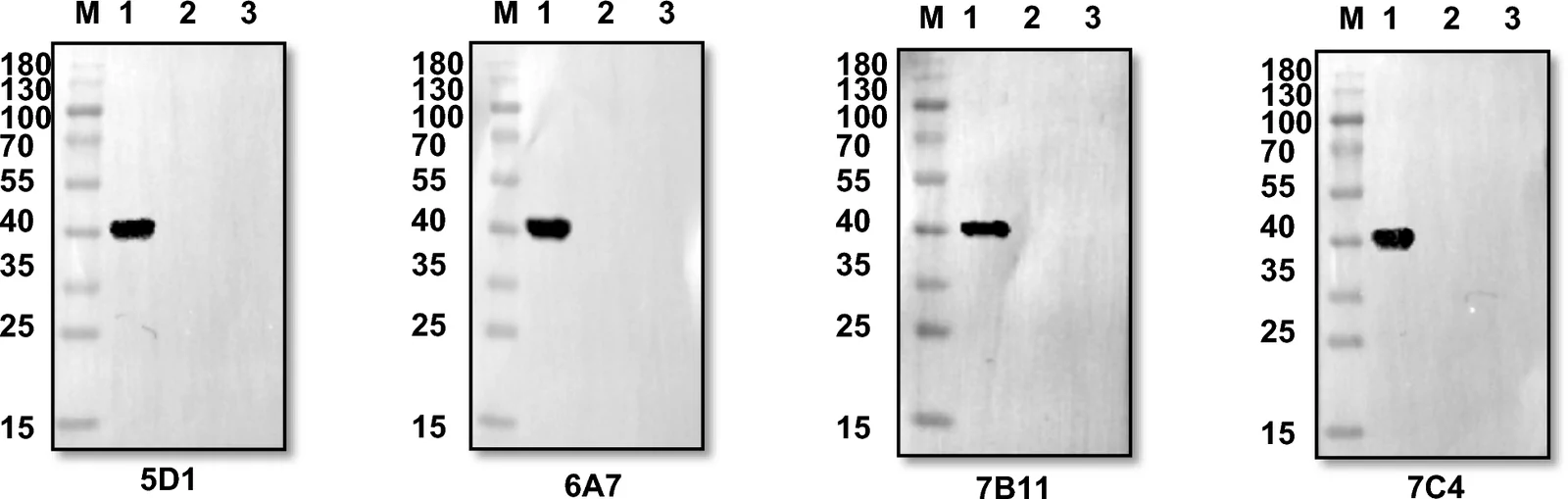
**Cross-reactivity analysis by western blotting.** The mAbs 5D1, 6A7, 7B11, and 7C4 were tested against recombinant VP7 proteins of EHDV-1, AHSV-1, and BTV-1 (lane 1: EHDV-1 VP7; lane 2: BTV-1 VP7; lane 3: AHSV-1 VP7). All four mAbs specifically recognized EHDV VP7 protein, with no cross-reactivity observed against AHSV or BTV VP7 proteins.

To determine the conservation of the identified antigenic epitopes within *Orbivirus*, the amino acid sequences of VP7 proteins from EHDV, BTV, and AHSV were aligned and analyzed. The results showed that the three linear epitopes ^83^DYIQNLATIGVLATPEI^99^, ^121^PDRQPFGYFL^130^, and ^229^APVNVNNPGQ^238^ were conserved across most EHDV serotypes, with only a single substitution at residue 130 in EHDV-4 and EHDV-5. Although this substitution occurred, the substituted amino acid remained nonpolar, and the physicochemical properties of this region were not significantly altered. Further sequence alignment indicated that none of the three epitopes were conserved in the VP7 proteins of AHSV and BTV, suggesting their EHDV species specificity (Figure 5). Moreover, cross-reactivity assays confirmed that mAbs 5D1, 7B11, 7C4, and 6A7 recognized the EHDV VP7 protein, and did not react with VP7 proteins of AHSV or BTV, which was consistent with the epitope conservation analysis (Figure 6).

### Bioinformatic analysis

**Figure 7:**
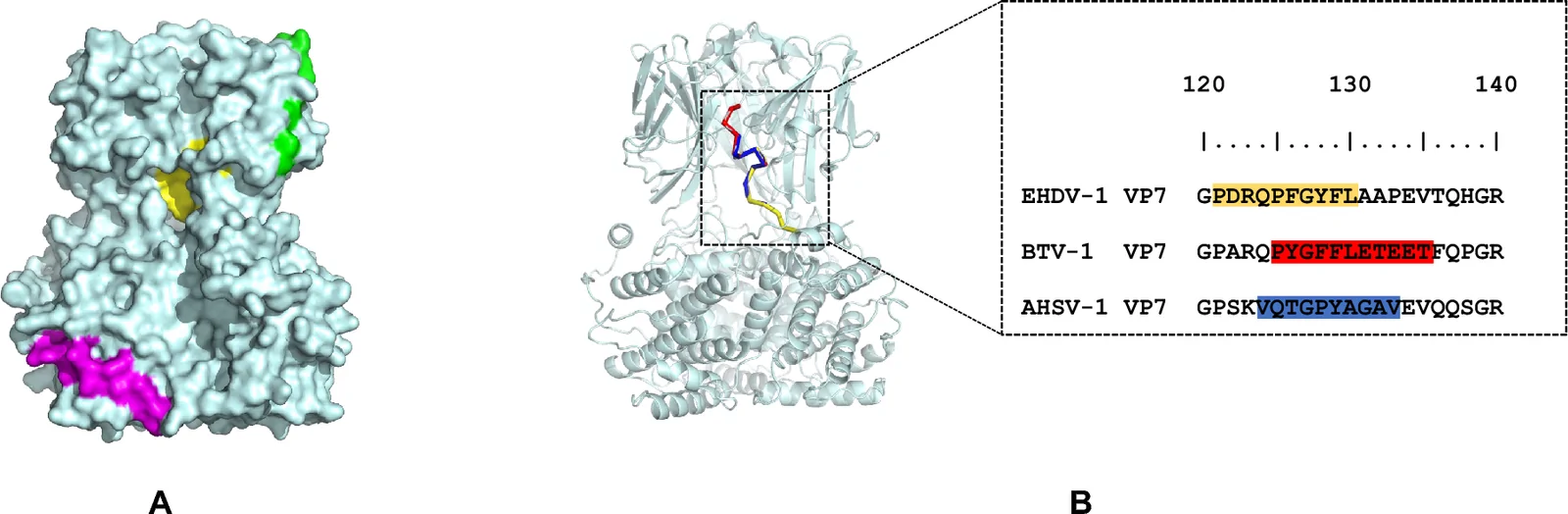
**Localization and three-dimensional spatial overlap of VP7 epitopes**. **A**: Localization of the epitopes recognized by mAbs 5D1, 6A7, 7B11, and 7C4 within the VP7 protein structure (palecyan: VP7 monomer, magenta: ^83^DYIQNLATIGVLATPEI^99^, yellow: ^121^PDRQPFGYFL^130^, and green: ^229^APVNVNNPGQ^238^). **B**: Three-dimensional spatial overlap of the antigenic VP7 epitopes of EHDV, BTV and AHSV. The epitopes of EHDV, BTV, and AHSV are shown in yellow, red, and blue, respectively.

The 3D structural model of EHDV-1 VP7 was predicted using SWISS-MODEL with 1BVP as the template. Structural analysis showed that the epitope ^83^DYIQNLATIGVLATPEI^99^ was located in the bottom domain, ^121^PDRQPFGYFL^130^ was located in the linker region between the bottom and top domains, and ^229^APVNVNNPGQ^238^ was located in the top domain (Figure 7A). Importantly, our laboratory previously identified linear B-cell epitopes located within the same linker region on the VP7 proteins of AHSV and BTV (AHSV VP7 124−132 aa and BTV VP7 125−135 aa, respectively) [43, 44]. Although the amino acid sequences of these three epitopes were not identical, their spatial positions within the three-dimensional structure of VP7 largely overlapped (Figure 7B). This linker region adopted a random coil conformation and was highly exposed and flexible, making it easily recognized by antibodies. Thus, this region became one of the common dominant epitope regions shared by the three viruses.

## Discussion

EHDV and BTV both belong to the genus *Orbivirus* within the family *Sedoreoviridae*. They share high similarities in transmission routes, host ranges, clinical symptoms, and viral morphology, posing great challenges to the differential diagnosis of the two viruses [45, 46]. The VP7 protein is a group-specific antigen with a highly conserved sequence between viruses, making it a common target for developing diagnostic assays [47]. However, due to the high amino acid sequence similarity of VP7 protein between EHDV and BTV serological detection methods based on this protein, such as indirect ELISA and agar gel immunodiffusion (AGID), are prone to erroneous results caused by cross-reactivity [48].

In traditional serological diagnosis, intact viruses or full-length recombinant proteins are commonly used as coating antigens. However, the cross-reactivity that frequently occurs among different pathogens directly compromises the specificity of detection methods, representing a key technical bottleneck in the advancement of serological diagnostics [49]. In contrast, diagnostic methods developed based on specific B-cell epitopes can effectively avoid the cross-reactivity and fundamentally enhance both specificity and reliability at the antigen level. In the diagnosis of dengue virus (DENV), the synthetic peptide P14M, obtained through screening with the DENV-1 specific mAb 15F3-1, can specifically recognize patients infected with DENV-1, with a sensitivity of 95% and a specificity of 100% [50]. In the field of hepatitis virus diagnosis, researchers prepared the specific mAbs 16D9 and 14A7 targeting the N-terminus and C-terminus of hepatitis Be antigen (HBeAg), respectively, and established an HBeAg detection method that effectively eliminated cross-reactivity, thus, significantly improving the accuracy of antigen detection [51]. The strategy of multi-epitope recombinant proteins has also been applied to the detection of hepatitis C virus [52, 53]. Those findings demonstrate that diagnostic approaches relying on B-cell epitopes improved diagnostic specificity and reliability, providing important technical support for precision diagnostics.

Although epitope-based methods have obvious advantages, whether there are shared epitopes between EHDV and BTV and how to distinguish them remain key issues that need to be clarified. One study immunized BALB/c mice with recombinant VP7 protein and obtained nineteen mAbs, of which fifteen specifically recognized EHDV VP7 protein and four recognized BTV VP7 protein [54]. Another study immunized BALB/c mice with EHDV-2 recombinant VP7 protein and generated sixty-six mAbs, of which fifty-five specifically recognized EHDV VP7 protein, thirteen cross-reacted with BTV VP7 protein, but none showed cross-reactivity with AHSV VP7 protein. Twenty of these mAbs showed potential for the development of a competitive ELISA [55]. Our previous work also demonstrated that four out of six mAbs against BTV VP7 cross-reacted with EHDV VP7 protein [43]. Collectively, the findings confirmed the presence of shared antigenic epitopes capable of eliciting antibody responses between the VP7 proteins of EHDV and BTV. Furthermore, by aligning the VP7 amino acid sequences of the *Orbivirus* members AHSV, BTV, and EHDV, and constructing a consensus sequence of the three viruses, eleven potential linear B-cell epitope regions and several discontinuous B-cell epitopes were predicted on the VP7 protein. These predictions provided a structural biology basis for elucidating the molecular basis of the serological cross-reactivity between EHDV and BTV [56]. The results collectively indicated that the VP7 proteins of EHDV and BTV immunologically shared common epitopes, providing a foundation for subsequent epitope identification and the development of specific diagnostic reagents.

In this study, BALB/c mice were immunized with recombinant VP7 protein expressed in *E. coli*. Four mAbs against EHDV VP7 protein were prepared by hybridoma technology and identified by ELISA, western blot, and indirect immunofluorescence assays. To identify the precise antigenic epitopes recognized by the four mAbs, the VP7 protein was truncated and expressed as a series of GST fusion proteins. Western blot analysis revealed three distinct antigenic epitopes: ^83^DYIQNLATIGVLATPEI^99^, ^121^PDRQPFGYFL^130^, and ^229^APVNVNNPGQ^238^. Homology analysis showed that all three epitopes were conserved among EHDV serotypes, but were not conserved in AHSV and BTV. Cross-reactivity assays further confirmed that all four mAbs specifically recognized EHDV VP7 protein without cross-reactivity with AHSV or BTV VP7 proteins. Those results indicated that the three B-cell epitopes identified in this study were specific to EHDV VP7, as their amino acid sequences differed from the corresponding regions of BTV VP7. The sequence divergence likely explains why the four mAbs generated in this study recognized EHDV VP7 specifically and did not cross-react with BTV VP7. These specific B-cell epitopes and their corresponding mAbs represented key tools for developing competitive ELISA-based diagnostic methods for EHDV. However, the diagnostic utility of the identified epitopes and mAbs, as well as the protective efficacy of epitope-based vaccines, requires further validation.

In summary, we successfully prepared four mAbs against the EHDV VP7 protein and identified three novel linear B-cell epitopes. The epitopes are highly conserved among different EHDV serotypes and exhibit no cross-reactivity with the VP7 proteins of AHSV or BTV. The findings provide a molecular basis for the development of epitope-based diagnostic techniques and vaccines for EHDV.

## Data Availability

No datasets were generated or analysed during the current study.
